# Supplementary material for: Ketogenic Diet Protects from Experimental Colitis in a Mouse Model Regardless of Dietary Fat Source
Source: Nutrients. 2024 Apr 29;16(9):1348. doi: 10.3390/nu16091348 (PMC11085069; doi:10.3390/nu16091348)
Supplement: Supplementary file 1 [file nutrients-16-01348-s001.zip › nutrients-2947988-supplementary.pdf]

**Supplementary material**

**Table S1.** Formulations of study diets. CD = control diet, SFA-KD = ketogenic diet with saturated fatty acids, LA-KD = ketogenic diet with linoleic acid.

| | **CD** | **SFA-KD** | **LA-KD** |
|---|---|---|---|
| | TD.220115 | TD.220117.PWD | TD.220116.PWD |
| **Ingredient** | **g/kg** | **g/kg** | **g/kg** |
| Casein | 100.0 | 180.0 | 180.0 |
| DL-Methionine | 1.6 | 2.88 | 2.88 |
| Corn Starch | 512.46 | 0 | 0 |
| Sucrose | 100.0 | 0 | 0 |
| Maltodextrin | 155.0 | 0 | 0 |
| Vegetable Shortening | 25.0 | 0 | 590.0 |
| Safflower Oil | 25.0 | 0 | 85.0 |
| Cellulose | 44.648 | 70.206 | 70.164 |
| Mineral Mix, w/o Ca & P (98057) | 13.4 | 24.8 | 24.8 |
| Calcium Phosphate, dibasic | 10.1 | 18.6 | 18.6 |
| Calcium Carbonate | 5.0 | 9.4 | 9.4 |
| Vitamin Mix, w/o choline, A, D, E (83171) | 5.0 | 13.875 | 13.875 |
| Choline Bitartrate | 2.5 | 4.625 | 4.625 |
| Vitamin E, DL-alpha tocopheryl acetate (500 IU/g) | 0.225 | 0.41 | 0.41 |
| Vitamin A, palmitate (500,000 IU/g) | 0.027 | 0.008 | 0.05 |
| Vitamin D3, cholecalciferol (50,000 IU/g in sucrose) | 0.03 | 0.056 | 0.056 |
| TBHQ, antioxidant | 0.01 | | 0.14 |
| Anhydrous Milkfat | | 675.0 | |
| Ethoxyquin, antioxidant | | 0.14 | |

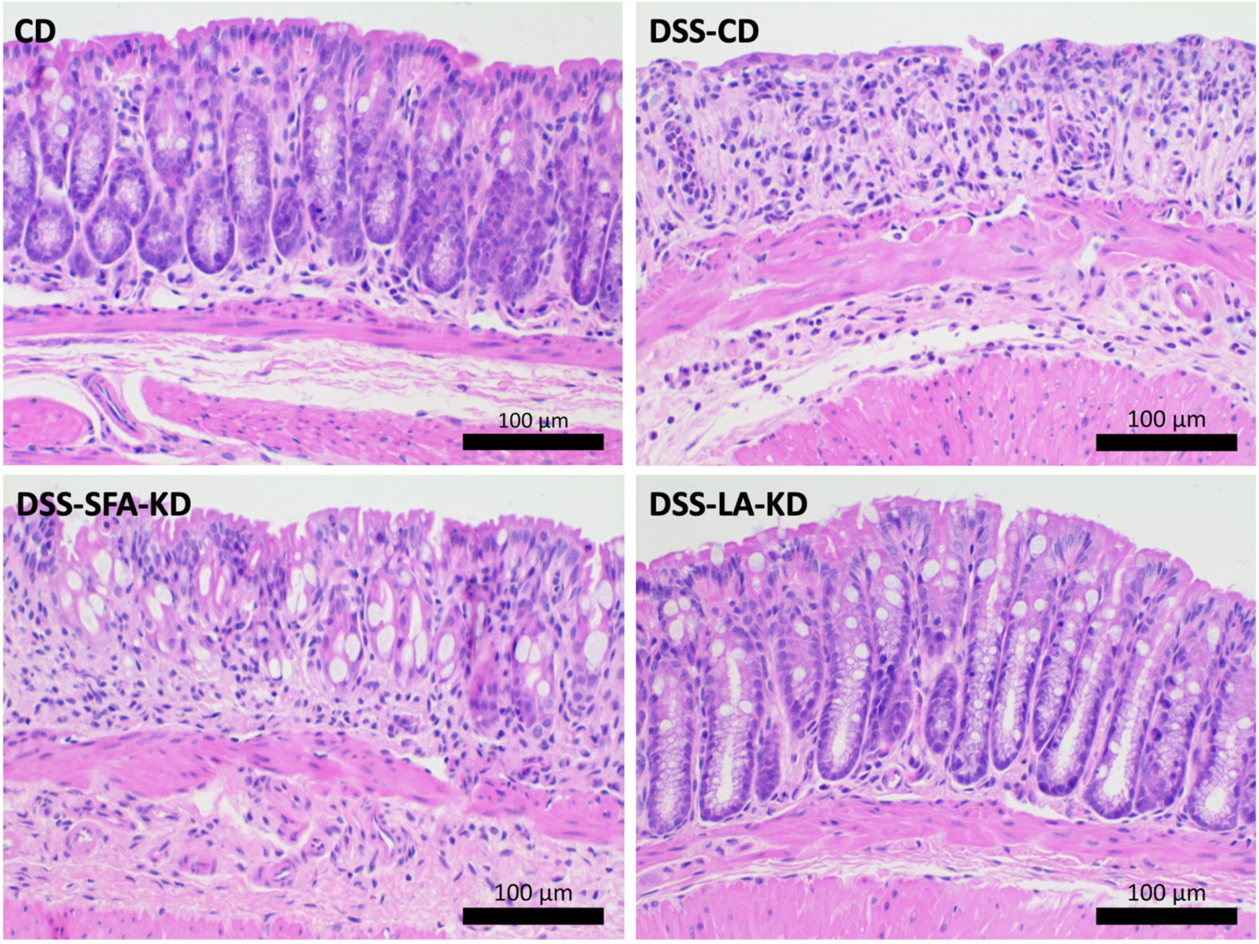


**Figure S1.** Original microphotographs of HE-stained colon sections. CD = healthy group with control diet, DSS-CD = DSS group with control diet, DSS-LA-KD = DSS group with diet high in linoleic acid, DSS-SFA-KD = DSS group with diet high in saturated fatty acids, HE = hematoxylin and eosin.

**A** 

## Claudin-1 and occludin

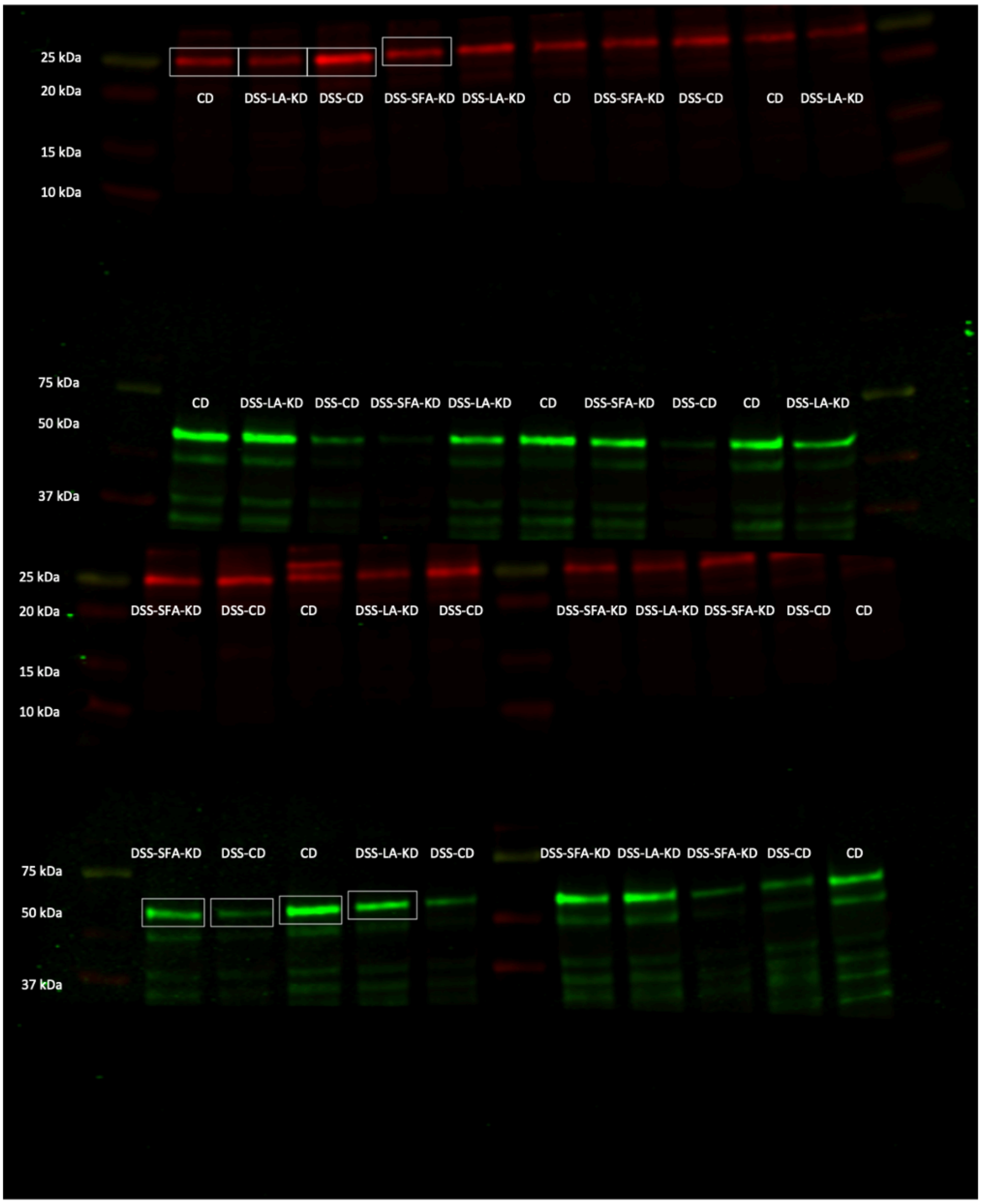

# Total protein for claudin-1 and occludin

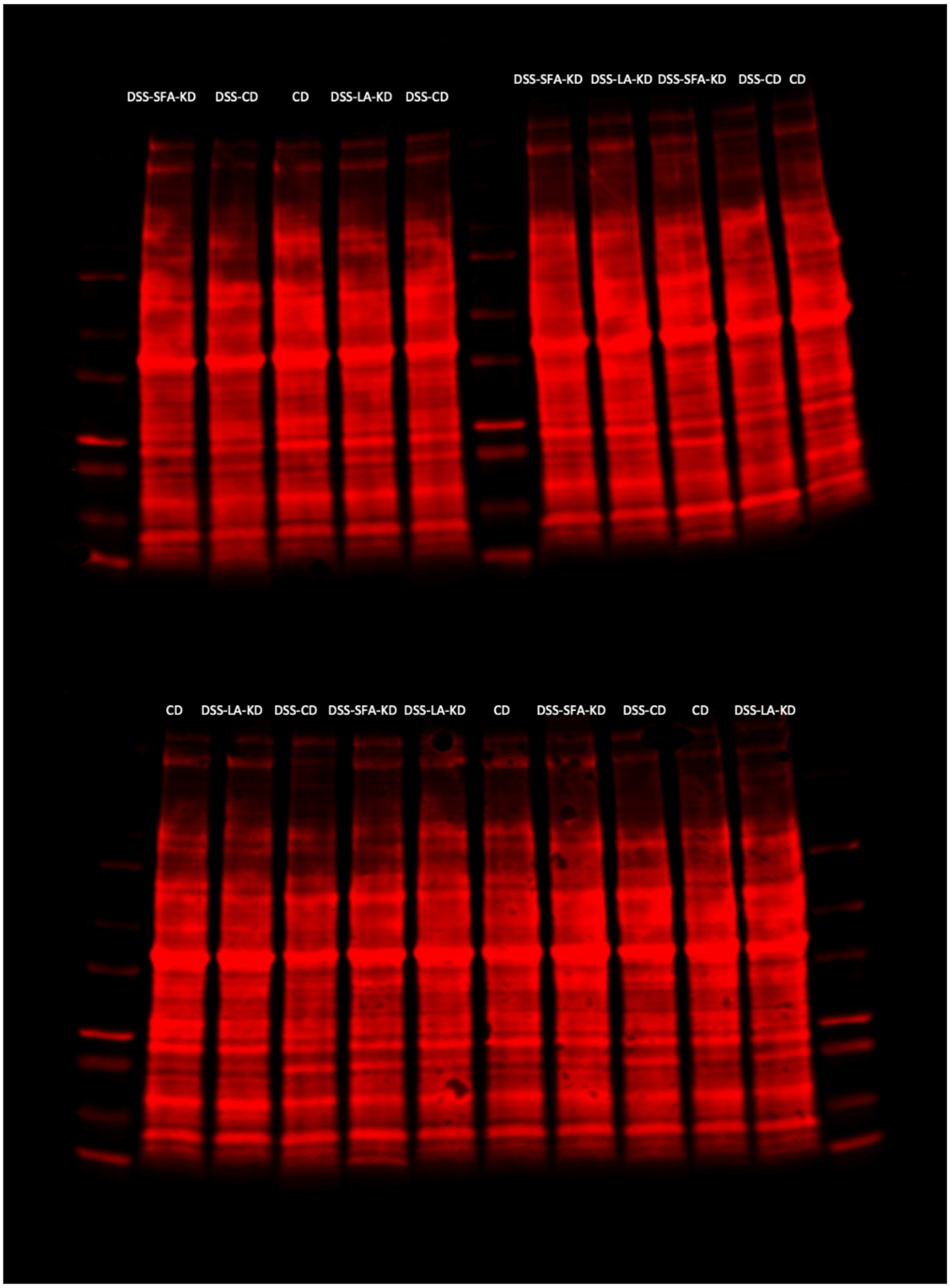

**B**

## Claudin-2

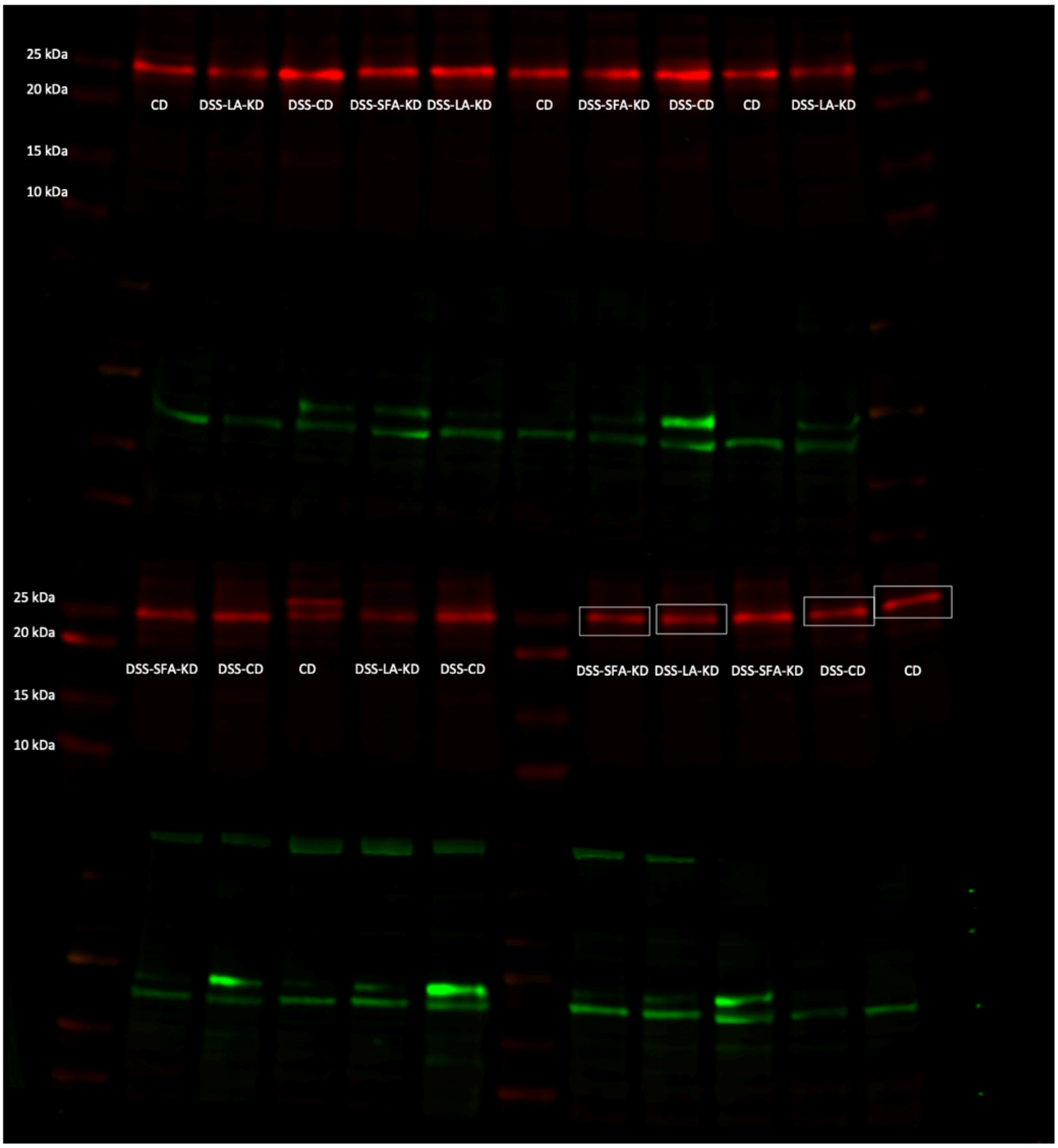
25 kDa
20 kDa
15 kDa
10 kDa
CD
DSS-LA-KD
DSS-CD
DSS-SFA-KD
DSS-LA-KD
CD
DSS-SFA-KD
DSS-CD
CD
DSS-LA-KD
25 kDa
20 kDa
15 kDa
10 kDa
DSS-SFA-KD
DSS-CD
CD
DSS-LA-KD
DSS-CD
DSS-SFA-KD
DSS-LA-KD
DSS-SFA-KD
DSS-CD
CD

# Total protein for claudin-2

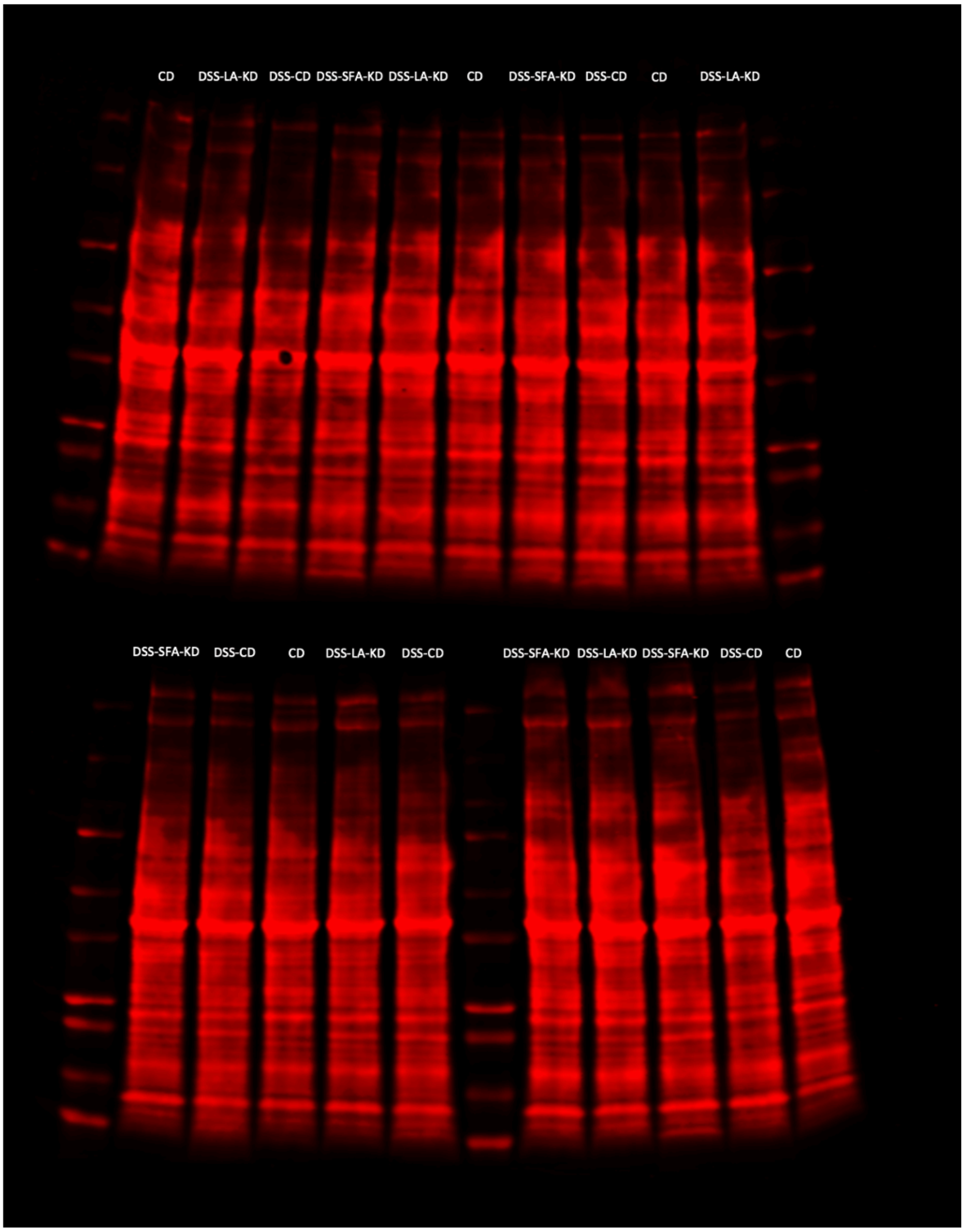

**C**

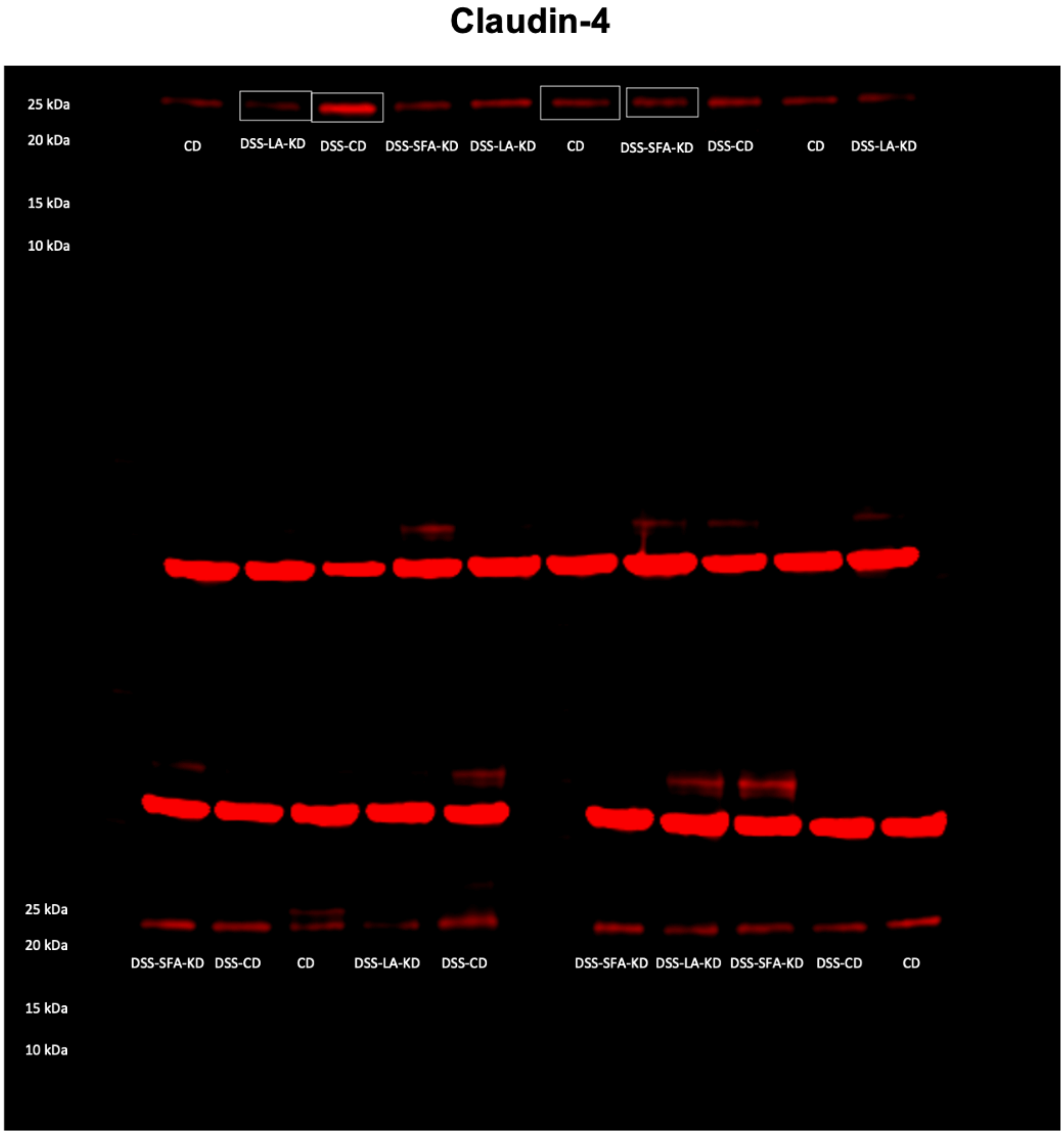
Claudin-4
25 kDa
20 kDa
15 kDa
10 kDa
CD
DSS-LA-KD
DSS-CD
DSS-SFA-KD
DSS-LA-KD
CD
DSS-SFA-KD
DSS-CD
CD
DSS-LA-KD
25 kDa
20 kDa
15 kDa
10 kDa
DSS-SFA-KD
DSS-CD
CD
DSS-LA-KD
DSS-CD
DSS-SFA-KD
DSS-LA-KD
DSS-SFA-KD
DSS-CD
CD

## Total protein for claudin-4

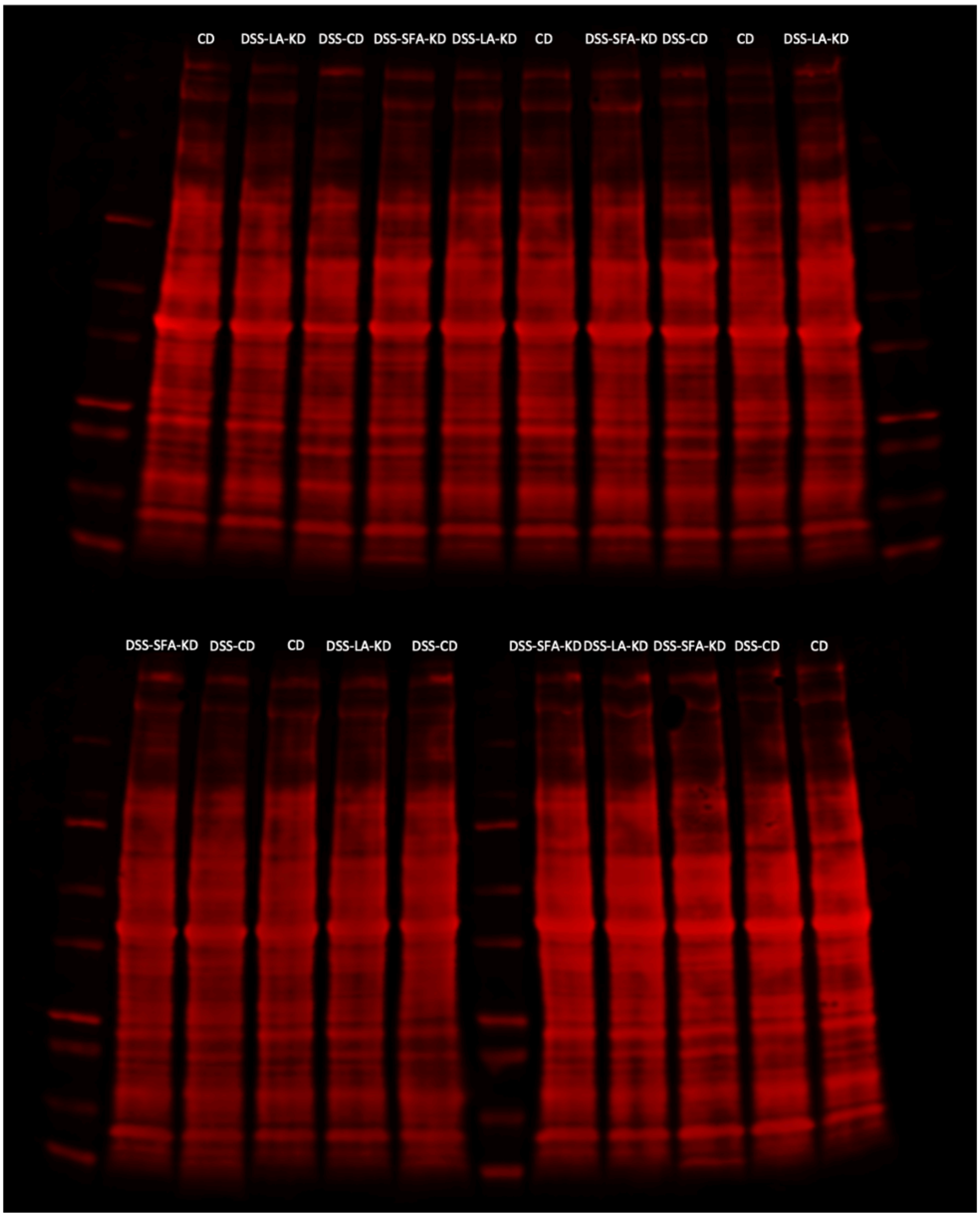


**Figure S2.** Original and unmodified Western Blot images of colonic tight junction proteins. A. Claudin-1 and occludin, B. claudin-2, C. claudin-4 and total protein. The bands presented in the manuscript are circled. CD = healthy group with control diet, DSS-CD = DSS group with control diet, DSS-LA-KD = DSS group with diet high in linoleic acid, DSS-SFA-KD = DSS group with diet high in saturated fatty acids.
